# Evolving role of myocardial fibrosis in heart failure with preserved ejection fraction

**DOI:** 10.3389/fcvm.2025.1573346

**Published:** 2025-04-23

**Authors:** Muhammad K. Malik, Menhel Kinno, Max Liebo, Mingxi D. Yu, Mushabbar Syed

**Affiliations:** ^1^ Department of Internal Medicine, Loyola University Medical Center, Maywood, IL, United States; ^2^ Department of Cardiology, Baylor Scott & White, The Heart Hospital, Plano, TX, United States; ^3^ Division of Cardiology, Department of Internal Medicine, Loyola University Medical Center, Maywood, IL, United States; ^4^ Division of Cardiology, Department of Internal Medicine, Rush University Medical Center, Chicago, IL, United States

**Keywords:** heart failure with preserved ejection, myocardial fibrosis, inflammation, biomarkers, collagen metabolism

## Abstract

Heart failure with preserved ejection fraction (HFpEF) is a complex clinical diagnosis with a heterogeneous pathophysiology and clinical presentation. The hallmark of HFpEF is diastolic dysfunction associated with left ventricular remodeling and fibrosis. Myocardial interstitial fibrosis (MIF) occurs as the result of collagen deposition and is dependent on the underlying etiology of heart failure. Detection of MIF can be done by invasive histopathologic sampling or by imaging. More recently, novel biomarkers have been investigated as an alternative tool for not only the detection of MIF but also for the prognostication of patients with HFpEF which may in turn alleviate the need for invasive and expensive imaging in the future.

## 1 Introduction

Heart failure remains a major cause of morbidity and mortality, with an estimated annual incidence of 960,000 new cases just in the United States of America. The incidence of heart failure with preserved ejection fraction (HFpEF) specifically continues to rise and now accounts for over 50% of all heart failure patients (1, 2). Patients with HFpEF are likely to have more comorbidities and heterogeneous symptoms than those with reduced ejection fraction (3). The complexity of HFpEF has led to the exploration of different markers to help diagnose, prognosticate, and manage this condition. Myocardial interstitial fibrosis (MIF), has been found to play an integral role in the development of diastolic dysfunction and can be detected through both invasive and non-invasive measures including biopsy, cardiac imaging, and novel biomarkers.

## 2 Diagnosis and presentation of HFpEF

According to the ACC/AHA/HFSA guidelines, the diagnosis of HFpEF requires a LVEF > 50% with evidence of spontaneous or provokable increased left ventricle filling pressures which can be assessed by biomarkers, non-invasive hemodynamics, or invasive hemodynamics (4). These criteria are sometimes challenging to apply to the HFpEF population. Obesity is a comorbidity associated with HFpEF and has been demonstrated to be a population in which b-type natriuretic peptide (BNP) is falsely low (5). Some HFpEF will only manifest imaging or hemodynamic evidence of cardiac congestion during exercise, and such invasive testing is not readily available everywhere (6).

## 3 Pathophysiology of HFpEF

The hallmark of HFpEF is diastolic dysfunction, which is most simply defined as inadequate filling of the left ventricle for a given preload. Abnormalities in the early or late phases of diastole, passive filling, and atrial contraction can lead to diastolic dysfunction.

Diastolic dysfunction can be a manifestation of epicardial or microvascular coronary disease, hypertension, diabetes, obesity, atrial fibrillation, and renal dysfunction (2, 7). These pathologies can lead to abnormalities in excitation-contraction coupling, left ventricular wall thickness, and myocardial stiffness; all of which over time can lead to decreased ventricular filling and elevated end-diastolic pressures (8, 9).

Extracardiac inflammatory states such as hypertension cause ventricular hypertrophy, and diabetes and obesity lead to elevated levels of pro-inflammatory cytokines (10). Intracardiac inflammation can occur from cardiomyocyte death in the setting of myocardial infarction leading to fibrosis (11). Fibrosis can also be the result of coronary microvascular endothelial dysfunction and systemic inflammation (12). Importantly, collagen deposition leading to fibrosis has been shown to increase myocardial stiffness thereby leading to the development of diastolic dysfunction and HFpEF (13).

## 4 Prognostication in HFpEF

Efforts have been made to define different phenotypes of HFpEF which may have different prognoses and treatment profiles (14). Phenotypes have been proposed based on subgroup analyses of previous clinical trials focusing on targeted drug therapy such as I-PRESERVE (15), CHARM-Preserved (16), and TOPCAT (17). Characteristics associated with a worse prognosis were patients older in age, higher comorbidity burden, and patients with elevated inflammatory biomarkers (14, 18, 19). With increasing comorbidity burden there is more systemic inflammation leading to more endothelial dysfunction and remodeling which over time lead to worse outcomes (14).

The HFA-PEFF (Heart Failure Association pre-test assessment, echocardiography and natriuretic peptide, functional testing, and final aetiology) and H_2_FPEF are scoring systems used to assist with the diagnosis of HFpEF (20, 21). Sub-studies of TOPCAT suggest that these scoring systems may also have prognostic implications with higher scores correlating with increased cardiovascular events (22). Machine learning algorithms are now being increasingly used to generate new HFpEF phenotypes and to create new scoring systems for prognostication (23, 24).

Advances in cardiac imaging have led to the use of “imaging biomarkers”. Furthermore, the development of novel laboratory biomarkers has been an increasing area of research for prognostication in HFpEF. For this review, we will focus on biomarkers of myocardial remodeling, specifically markers of fibrosis and extracellular matrix (ECM) remodeling as they relate to MIF.

## 5 Myocardial fibrosis in HFpEF

Myocardial fibrosis occurs due to collagen deposition in the myocardial interstitial space in response to intrinsic cardiac injury and systemic neurohormonal changes (25). Reparative, or focal, myocardial fibrosis is either subendocardial or transmural and is most commonly seen in the setting of myocardial infarction whereas reactive, or diffuse, interstitial fibrosis occurs in the mid-wall or subepicardium and is seen in the setting of non-ischemic cardiomyopathy (NICM) (26). The process of fibrosis begins with the activation of cardiac fibroblasts in response to injury of the cardiac myocytes. Cardiac fibroblasts in turn induce the activation of ECM proteins and cytokines creating a profibrotic secretome that ultimately leads to ECM remodeling and collagen deposition (25, 27). Type I collagen is the predominant form of collagen found in NICM fibrosis and is associated with increased myocardial stiffness and diastolic dysfunction (27). MIF is clinically relevant as the degree of collagen deposition present in addition to the types of collagen present leads to myocardial stiffness which in turn leads to diastolic dysfunction.

Not only is the total amount of collagen burden, or collagen volume fraction (CVF), important but also how endomysial and perimysial collagen is arranged microscopically can influence myocardial mechanics (25, 28). Some studies have suggested that the CVF did not have any correlation with ejection fraction (28, 29) further proposing that the qualitative aspect of collagen deposition is more integral in the pathophysiology of HFpEF. An example of such is demonstrated by higher levels of collagen cross-linking being associated with increased severity of left ventricle diastolic dysfunction (30). Higher levels of fibrosis have also been associated with an increased risk of hospitalization, cardiac events, and death in patients with heart failure (31, 32).

## 6 Detection of myocardial fibrosis

### 6.1 Myocardial biopsy

The gold standard of diagnosing MIF is with endomyocardial biopsy. Histologically, fibrosis is visualized by the presence of micro scars which are focal areas of fibrosis often seen with ischemia, or by thick sheaths and bands that surround the perivascular space and muscle bundles (25). While tissue sampling can provide a definitive answer regarding the type of collagen present and the collagen burden; the means of obtaining such a sample are invasive and carry risk. Additionally, sampling error may reduce accuracy as biopsy samples are taken from the right ventricle as opposed to the left ventricle where majority of the pathology occurs.

### 6.2 Cardiovascular magnetic resonance imaging

**Figure 1:**
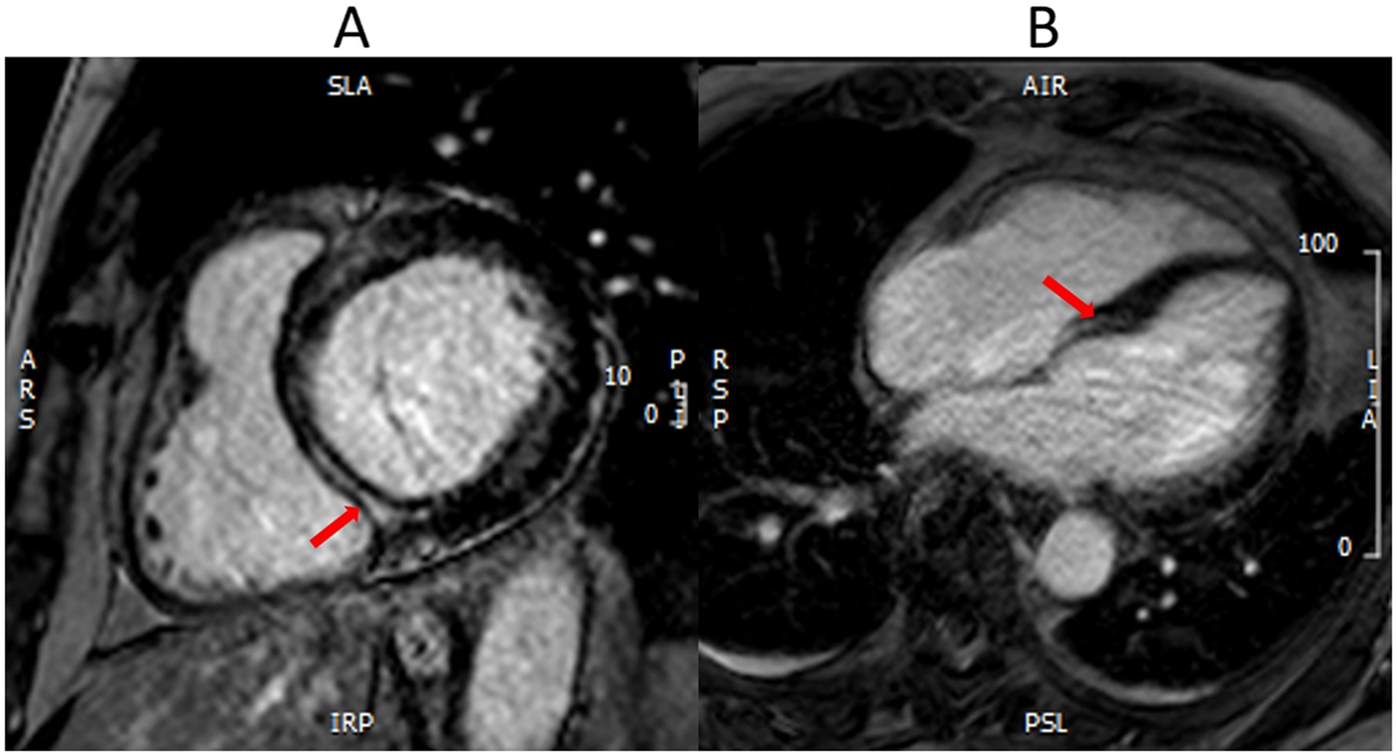
Myocardial fibrosis with CMR. CMR LGE demonstrates a white stripe (red arrows) in the mid-wall of basal inferoseptum in the short axis view **(A)** and four-chamber view **(B)**. This mid-wall fibrosis is consistent with the non-ischemic scar pattern seen in non-ischemic cardiomyopathy.

**Figure 2:**
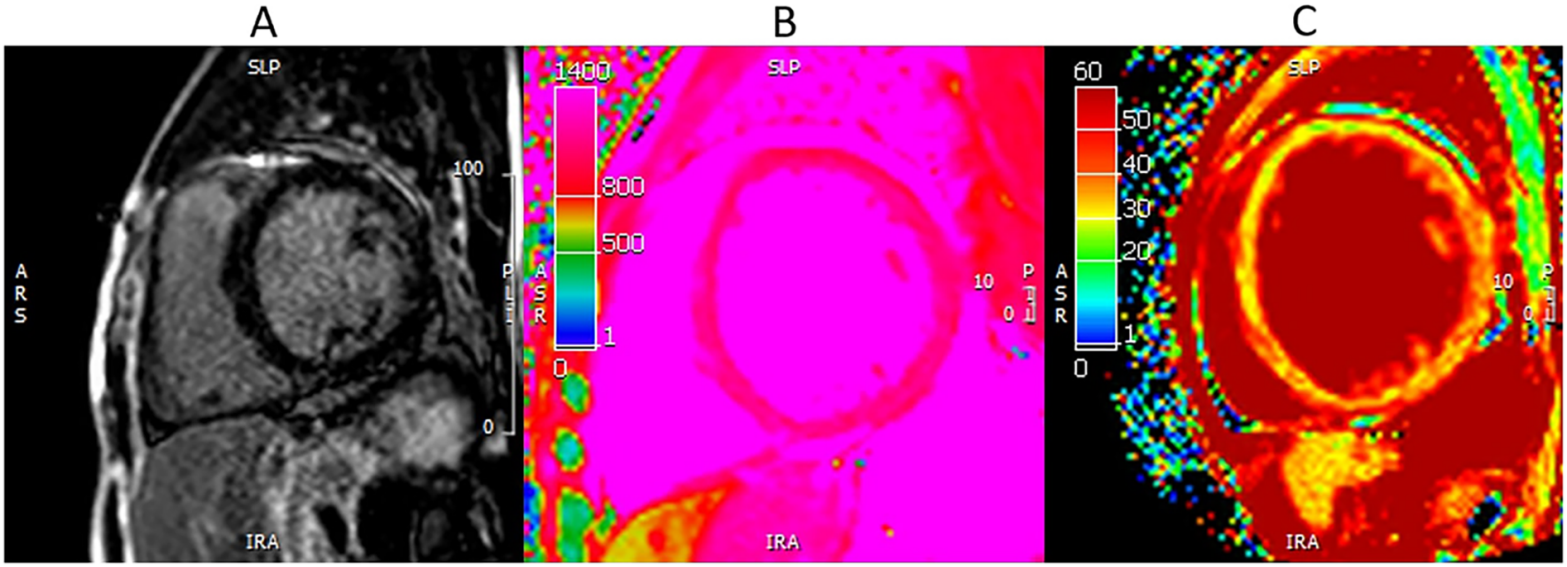
LGE, T1 mapping, ECV on CMR. A 56-year-old male with non-ischemic cardiomyopathy. CMR-LGE **(A)** showed no visible fibrosis; however, T1 mapping **(B)** and ECV **(C)** are elevated consistent with interstitial fibrosis (T1 = 1,150 ms, ECV 31%).

CMR is being increasingly utilized as a well-validated technique for the assessment of scar and fibrosis (33). CMR utilizes late gadolinium enhancement (LGE), native T1 mapping, and post contrast T1 mapping for extracellular volume fraction (ECV) to assess scar burden, diffuse fibrosis, and extracellular space (Figures 1, 2).

LGE depicts the relative difference in longitudinal recovery times between gadolinium-enhancing areas of fibrosis and normal myocardium (34). LGE is particularly useful as it differentiates between various etiologies of fibrosis depending on the pattern and location. Scar burden detected by LGE was independently predictive of repeat hospitalizations, rates of ventricular tachycardia, and major adverse cardiac events (33, 35, 36). A limitation of LGE is that it may miss diffuse fibrosis since there is limited normal myocardial tissue to compare to (33, 34). Native T1 mapping provides a pixel-wide map of the myocardial T1 relaxation time that can help assess the degree of tissue edema, changes in the interstitial space, and detection of interstitial fibrosis (37). ECV is an excellent technique to study disease processes that expand the extracellular/interstitial space to detect interstitial fibrosis or infiltration and can also be used as a marker of tissue remodeling as collagen deposition will contribute to increases in ECV (37). T1 mapping and ECV provide quantitative values that can be tracked over time or in response to treatment. ECV has also been demonstrated to be an independent predictor of repeat hospitalization and morality in HFpEF patients (33, 38). CMR remains limited by its availability, with other potentially limiting factors including the length of scan, claustrophobia, metallic hardware, and arrhythmias (39).

More recently, cardiac metabolic imaging has gained interest. Cardiac metabolism can be assessed by analyzing concentrations of myocardial metabolites using magnetic resonance spectroscopy (MRS) and hyperpolarized MRS (40). These two imaging modalities are primarily utilized in research settings and are not currently routinely used in the clinical setting.

### 6.3 Cardiac computed tomography

Cardiac CT is an alternative modality to CMR for the evaluation of LV structure and myocardial tissue characterization according to the 2022 ACC/AHA guidelines (41). The use of cardiac CT was validated for use in detecting non-ischemic fibrosis based on a study by Langer et al. in 2014 (42). The main limitations of cardiac CT include a large amount of iodine contrast administration low signal-to-noise ratio, and need for dedicated protocols (39). Advances in cardiac CT imaging include the use of dual-energy and photon counting which may address some of these limitations but need further research.

### 6.4 Positron emission tomography and single-photon emission computed tomography

Positron emission tomography (PET) and single-photon emission computed tomography (SPECT) are two forms of nuclear imaging used for the assessment of myocardial perfusion (43). Fibrosis can be indirectly detected as areas of irreversible perfusion defects (44). With PET, the perfusable tissue index is an indirect marker of fibrosis (reduced index correlating to increased fibrosis), while a reduction in 18-fluorodeoxyglucose (18-FDG) can more specifically assess fibrosis (44, 45). Although limited by its availability, fibrosis can be more specifically assessed by SPECT with the use of technetium-99 labeled Cy5.5-RGD, an imaging peptide that is a marker of collagen deposition (44, 46). Both PET and SPECT have lower spatial resolution that can limit the identification of small scars or as lower degree of fibrosis. Like CMR and cardiac CT, the length of the study and cost/insurance approval are limitations.

### 6.5 Echocardiography

Echocardiography has limited utility in myocardial tissue characterization. Its main role comes in the evaluation of global longitudinal strain which has been demonstrated to estimate the amount of fibrosis present (47). More advanced forms of echocardiography including power modulation echocardiography and speckle-tracking echocardiography can also be used to assess myocardial scar burden (48). In a study by Papachristidis et al., they found good scar volume agreement between CMR and power modulation echocardiography, a specialized form of echocardiography using a non-linear beam with a contrast-enhancing agent to detect scar (49). With regards to speckle-tracking echocardiography, studies have demonstrated early detection of myocardial disease (50).

## 7 Biomarkers

Biomarkers have played an integral role in diagnosing, managing, and prognosticating patients with heart failure. BNP and NT-pro-BNP, are the two most common biomarkers used in routine clinical practice and are markers of myocardial stretch and have class I and II recommendations for the diagnosis and management of HFpEF. Additional categories of biomarkers include markers of myocardial injury, neurohormonal activity, and more recently markers of fibrosis and collagen metabolism (Table 1). In the 2017 ACC/AHA guidelines for risk stratification in patients with chronic heart failure, novel markers of myocardial remodeling [soluble suppression of tumorigenesis 2 (sST2) and galectin-3 (Gal-3)] were given a class IIb recommendation for use in clinical practice (51). The increasing amount of research and utilization of biomarkers may alleviate the need for the expensive and potentially hazardous imaging techniques discussed above.

**Table 1:** Biomarkers.

Biomarker	Domain	Clinical utility
NT-proBNP	Myocardial stretch	Class I and II recommendations for diagnosis of heart failure with preserved ejection fraction in 2013 ACC/AHA guidelines
sST2	Fibrosis	Class IIa level of evidence in 2017 ACC/AHA guidelines for heart failure risk stratification
Gal-3	Fibrosis	Class IIa level of evidence in 2017 ACC/AHA guidelines for heart failure risk stratification
MMP	Collagen degradation	MMP-2 and MMP-3 are associated with higher mortality in patients with heart failure
TIMP	Collagen synthesis	Elevated in heart failure patients; an independent predictor of all-cause death
PIIINP	Collagen synthesis	Associated with the development of heart failure; inversely proportional to survival in heart failure patients
CITP	Collagen degradation	Associated with the development of heart failure
PICP	Collagen deposition	Potentially prognostic given response to diuretic treatment
CITP:MMP-1	Collagen cross-linking	Higher amount of crosslinking and collagen deposition was associated with more heart failure hospitalizations

Biomarkers of myocardial fibrosis and collagen metabolism and their clinical implications.

### 7.1 Soluble suppression of tumorigenesis 2 (sST2*)*

Soluble suppression of tumorigenesis 2 (sST2) is a member of the cytokine interleukin-1 (IL-1) receptor family and is released under conditions of myocardial strain and cardiovascular injury (52). sST2 levels are elevated immediately post myocardial infarction (53). sST2 functions as a decoy receptor for the IL-33, the ligand for suppression of tumorigenesis 2 (ST2), therefore inhibiting the binding of ST2 to IL-33. The ST2/IL-33 interaction mediates an anti-fibrotic and anti-apoptotic response, thus sST2 induces a cascade that favors myocardial cell death, fibrosis, and remodeling (54).

The ability of sST2 to be used as a potential novel biomarker in heart failure was demonstrated in a study by Weinberg et al. in 2004 where they found that baseline sST2 and BNP were correlated, and the change in sST2 from baseline was a predictor of mortality in patients with NYHA functional class III-IV (55). Using samples from the PRIDE study (Pro-Brain Natriuretic Peptide Investigation of Dyspnea in the Emergency Department), sST2 was found to be strongly predictive of death at one year in patients with decompensated heart failure compared to NT-proBNP (56). In a study of 876 patients with a mean EF of 34%, sST2 and galectin-3 (an additional biomarker of inflammation and fibrosis) were independent predictors of hospitalizations (57).

In HFpEF specifically, a study using samples from the TOPCAT trial (Treatment of Preserved Cardiac Function Heart Failure with an Aldosterone Antagonist Trial) identified a dominant cluster of biomarkers of inflammation and fibrosis and found sST2 to be predictive of acute decompensated heart failure (58). Further studies have also demonstrated sST2 as a predictor of mortality and rehospitalization thirty days post-discharge in elderly HFpEF patients (59).

### 7.2 Galectin-3 (Gal-3)

Galectin-3 (Gal-3) is a macrophage lectin product that is involved with cell differentiation, inflammation, and fibrogenesis (60). It is released from a variety of human tissues including myocardial cells and can have a wide range of effects depending on its location in cells: cytoplasmic Gal-3 is involved in cell survival and nuclear Gal-3 is involved in gene transcription related to inflammation, fibrogenesis, and interactions with the ECM. For these reasons, Gal-3 has been implicated in the development of myocardial remodeling (61). Unlike natriuretic peptides, it is not affected by age, body mass index, or sex (62).

Gal-3 is elevated in patients with acute heart failure and has demonstrated promise as a prognostic marker as well (63). A meta-analysis by Chen et al. in 2015 looked at Gal-3 as a prognostic biomarker and showed that a one percent increase in Gal-3 was associated with a thirty percent increase in mortality (64). There is debate regarding the prognostic ability of Gal-3 in a systematic review by Srivatsan et al. which suggested that Gal-3 was not predictive after adjustment for eGFR and LVEF (65). Compared to sST2, Gal-3 may be inferior as a prognostic biomarker; however, the combination of both NT-proBNP and Gal-3 together was associated with better prediction of mortality (63, 66).

With HFpEF specifically, a prospective study by de Boer et al. showed that the prognostic ability of Gal-3 for the primary outcome all-cause mortality and heart failure hospitalizations was stronger in HFpEF patients compared to HFrEF patients although the overall levels of Gal-3 were similar in both groups (67). Gal-3 has been associated with increased diastolic dysfunction and the severity of left ventricular stiffness in HFpEF (67).

Limitations regarding the use of sST2 and Gal-3 clinically remain the limited availability of the enzyme-linked immunosorbent assay (ELISA) test needed to measure them. Additionally, there are analytical considerations regarding the heterogeneity of different ELISA tests, especially with sST2 (68). It is important to note that these biomarkers are not specific for heart failure thus they are not considered diagnostic biomarkers.

### 7.3 Matrix metalloproteinases (MMP) and tissue inhibitors of metalloproteinases (TIMP)

Matrix metalloproteinases (MMP) are a group of twenty-five enzymes that are involved in the process of ECM degradation. Tissue inhibitors of metalloproteinases (TIMP) inhibit MMP and thus mitigate ECM breakdown. The balance between MMPs and TIMPs collectively helps regulate the amount of ECM remodeling that occurs (69). MMP-1, MMP-8, MMP-13, and MMP-18 collectively are collagenases and cleave collagen I, II, and III; although the ability to cleave collagen is not exclusive to the collagenase MMPs. Chronic inflammatory states such as hypertension can lead to induction of MMP (70). Over time, however, the prolonged stimulation of MMP will induce TIMP leading to long-term ECM remodeling (71).

MMP-2 has been studied as a biomarker in HFpEF (70). In a study looking at patients with LV hypertrophy and diastolic dysfunction, MMP-2 was a predictor of HFpEF (72).

### 7.4 Procollagen type III N-terminal propeptide (PIIINP) and carboxy-terminal telopeptide of collagen type I (CITP)

Procollagen type III N-terminal propeptide (PIIINP) is a propeptide of collagen type III and reflects collagen synthesis and degradation (73). Carboxy-terminal telopeptide of collagen type I (CITP) is formed after cleavage of collagen type I that occurs during ECM remodeling and reflects collagen degradation (73). Collagen I and III, the two predominant forms of collagen in the heart, have been studied as potential biomarkers of collagen metabolism and ECM remodeling (74, 75).

The Cardiovascular Health Study found that collagen biomarkers, specifically PIIINP and CITP were associated with the development of heart failure (76) and in further studies have been associated with a higher risk of incident HFpEF as compared to HFrEF (73).

### 7.5 Carboxy-terminal propeptide of collagen type I (PICP) and CITP:MMP-1

As mentioned previously MIF is determined in part both by the amount of collagen deposition, CVF, and the way in which the collagen is deposited. Carboxy-terminal propeptide of collagen type I (PICP) is a marker of type I collagen and is produced during the conversion of procollagen type I into collagen type with levels correlating with the CVF, providing an alternative means of evaluating CVF in addition to CMR mentioned previously (26). Likewise, the CITP:MMP-1 ratio can be used to evaluate the amount of collagen cross-linking—an increased amount of crosslinking confers resistance to MMP-mediated collagen degradation leading to low CITP:MMP-1 (26, 77). Levels of PICP have been associated with increasing mortality rates in HFpEF (78). Interestingly, levels of PICP/CVF have been demonstrated to decrease in response to treatment with torsemide and spironolactone thus proposing that these markers may have prognostic implications (79, 80).

## 8 Clinical implications

Increasing levels of fibrosis are associated with worse outcomes; thus, MIF as a therapeutic target has been a topic of research. Novel biomarkers of fibrosis can aid in diagnosing and prognosticating patients with HFpEF. Additionally, these markers can be trended as a therapeutic target with medical therapy. Large-scale trials have been performed investigating the roles of medical therapy as they pertain to ECM/collagen biomarker metabolism. Suppression of the renin-angiotensin-aldosterone pathway has been shown to reduce and improve myocardial fibrosis (81). Mineralocorticoid blockade with spironolactone has been demonstrated to decrease levels of procollagen peptide biomarkers and has been associated with improved morbidity and mortality (82, 83). Angiotensin-converting enzyme inhibition with lisinopril was shown to have a statistically significant reduction in myocardial fibrosis as demonstrated by a reduction in CVF on endomyocardial biopsy at six months in patients with baseline fibrosis (81). While there is limited clinical data on the use of sacubitril-valsartan in this area, animal studies have shown a reduction in levels of fibrosis in mice with HFrEF (84). Further clinical data is needed about angiotensin receptor/neprilysin inhibition and MIF and there is currently a prospective trial, REVERSE-LVH investigating this (85). Animal studies have shown that sodium-glucose cotransporter 2 inhibitors (SGLT2i) mediate inflammation and decrease levels of collagen on biopsy (86).

## 9 Discussion

HFpEF continues to be a developing area of research given the heterogenous nature of this disease. MIF and diastolic dysfunction form the basis of developing HFpEF. Imaging of myocardial fibrosis has an evolving role in understanding the pathophysiology and prognosis of HFpEF. Future research looking at a collagen-targeted contrast agent may provide accurate and early detection of myocardial fibrosis. Novel biomarkers represent a large area of ongoing research and have the potential to become more clinically relevant with the addition of myocardial injury and fibrosis biomarkers to the ACC/AHA risk stratification guidelines. More research is needed to characterize myocardial fibrosis in HFpEF in addition to the development of targeted treatments.
